# NCX1/Ca^2+^ promotes autophagy and decreases bortezomib activity in multiple myeloma through non-canonical NFκB signaling pathway

**DOI:** 10.1186/s12964-024-01628-4

**Published:** 2024-05-06

**Authors:** Tingting Li, Pingping Xiao, Dongbiao Qiu, Apeng Yang, Qingjiao Chen, Junfang Lin, Yao Liu, Junmin Chen, Zhiyong Zeng

**Affiliations:** 1 Department of Hematology, The First Affiliated Hospital of Fujian Medical University, Fuzhou, China; 2 Department of Blood Transfusion, The First Affiliated Hospital of Fujian Medical University, Fuzhou, China; 3 Fujian Key Laboratory of Laboratory Medicine, Fuzhou, China; 4 Department of Hematology, National Regional Medical Center, Binhai Campus of the First Affiliated Hospital, Fujian Medical University, Fuzhou, China; 5 Chongqing Key Laboratory of Translational Research for Cancer Metastasis and Individualized Treatment, Department of Hematology-Oncology, Chongqing University Cancer Hospital, Chongqing, China

**Keywords:** Sodium-calcium exchanger 1, Calcium, Bortezomib, Autophagy, NFκB

## Abstract

Although bortezomib (BTZ) is the cornerstone of anti-multiple myeloma (MM) therapy, the inevitable primary and secondary drug resistance still seriously affects the prognosis of patients. New treatment strategies are in need. Sodium-calcium exchanger 1 (NCX1) is a calcium-permeable ion transporter on the membrane, and our previous studies showed that low NCX1 confers inferior viability in MM cells and suppressed osteoclast differentiation. However, the effect of NCX1 on BTZ sensitivity of MM and its possible mechanism remain unclear. In this study, we investigated the effect of NCX1 on BTZ sensitivity in MM, focusing on cellular processes of autophagy and cell viability. Our results provide evidence that NCX1 expression correlates with MM disease progression and low NCX1 expression increases BTZ sensitivity. NCX1/Ca^2+^ triggered autophagic flux through non-canonical NFκB pathway in MM cells, leading to attenuated the sensitivity of BTZ. Knockdown or inhibition of NCX1 could potentiate the anti-MM activity of BTZ in vitro and vivo, and inhibition of autophagy sensitized NCX1-overexpressing MM cells to BTZ. In general, this work implicates NCX1 as a potential therapeutic target in MM with BTZ resistance and provides novel mechanistic insights into its vital role in combating BTZ resistance.

**Supplementary Information:**

The online version contains supplementary material available at 10.1186/s12964-024-01628-4.

## Introduction

Multiple myeloma (MM) is the second most common hematological malignancy, characterized by abnormal proliferation of malignant plasma cells in the bone marrow (BM) [1]. Clinically, it is often accompanied by bone destruction, hypercalcemia, anemia, osteolytic fracture and renal dysfunction [2]. Although the combination of proteasome inhibitors (PI), immunomodulators and monoclonal antibodies has significantly improved the survival rate of MM patients, relapse due to the first-in-class PI bortezomib (BTZ)- resistance is inevitable, and the disease remains incurable [3]. Therefore, the identification of effective targets to enhance BTZ sensitivity for MM treatment are urgently required.

Previous studies demonstrated that inhibition of autophagy significantly enhanced the efficacy of many anti-cancer drugs [4–6], and in vitro models, synergistically increased the cytotoxicity of BTZ in myeloma [7] and liver cancer [8]. In addition, a phase I clinical trial showed that the combination of hydroxychloroquine (a well-known autophagy inhibitor) and BTZ is an effective treatment for patients with relapsed/refractory myeloma [9]. In view of the previous studies, inhibition of autophagy in myeloma may be an important way to reverse the drug resistance phenotype and increase BTZ sensitivity.

Ca^2+^, as a second messenger, is able to regulate autophagy by activating or inactivating various calcium ion channels [10]. In recent studies, downregulation of transient receptor potential vanilloid 4 (TRPV4), a membrane calcium transporter, has been shown to inhibit autophagy in MM [11]. Interestingly, disturbance of cellular calcium homeostasis has also been reported to be closely related to the sensitivity of myeloma to BTZ [12, 13]. Although the roles of calcium transporters and autophagy in the development of MM have attracted increasing attention in recent years, their detailed molecular mechanisms in MM drug sensitivity have not yet been elucidated.

The Na^+^-Ca^2+^ exchanger 1 (NCX1) is a ubiquitously expressed plasma membrane bidirectional transporter, which regulates intracellular Ca^2+^ homeostasis [14]. Previous research data indicate that NCX1 plays different roles in different cancers. It is highly expressed in esophageal squamous cell carcinoma [15], human hepatocellular carcinoma [16] and prostate cancer [17], and mainly has a reverse transport mode. In glioblastoma cells, the forward transport mode is dominant [18, 19]. And there are different regulations in different breast cancer types [20]. Our preliminary data indicate that NCX1 is highly expressed in MM and exhibits a predominantly reverse transport mode. Furthermore, we determined that NCX1 had a significant impact on the viability of MM cells, indicating that NCX1 is a potential therapeutic target in MM. And NCX1 expression were positively correlated with serum calcium in MM. More importantly, NCX1 induced calcium influx of MM cells promoted RANKL-induced osteoclast differentiation [21]. Studies have confirmed that the BM microenvironment, especially BM osteoclasts, play a central role in supporting MM cell survival and drug resistance [22, 23]. It is unclear whether NCX1 can affect the BTZ sensitivity of MM through osteoclasts . Therefore, in this study we will explore the role of NCX1 on BTZ sensitivity in MM and explore its underlying mechanism.

## Materials and methods

### Clinical specimens and patients

We collected paraffin sections of bone marrow (BM) specimens from newly diagnosed MM patients in the First Affiliated Hospital of Fujian Medical University from September 2019 to December 2022. These patients were diagnosed according to the guidelines for the diagnosis and management of multiple myeloma in China [24].

Inclusion criteria for MM patients: receiving two or more courses of BTZ treatment, age≤75 years old. Exclusion criteria: not receiving BTZ treatment; receiving less than two courses of BTZ treatment; age > 75 years old; lost to follow-up during the follow-up period; unable to receive regular standardized chemotherapy. MM patients were divided into 3 stages according to the International Staging System (ISS) [25], and the correlation between NCX1 expression and ISS stage of MM was analyzed. The percentages of CD138+ cells in BM were obtained from pathology reports. Paraffin sections of BM samples from 18 patients with iron deficiency anemia (IDA) were collected as controls. In addition, informed consent was obtained in accordance with the Declaration of Helsinki and approved by the Ethics Committee of the First Affiliated Hospital of Fujian Medical University (number for approval:[2023]243).

### Cell culture

Human MM cell lines of RPMI8226 and KMS11 were purchased from Procell ( Catalogue number: CL-0564 and CL-0794). MM cell lines were maintained in RPMI 1640 medium containing 10% FBS (Supplier name: ExCell, China;Catalogue number: FND500), penicillin and streptomycin at 37 ℃ in a humidified atmosphere of 5% CO_2_ atmosphere. Cells were monitored for mycoplasma contamination using a mycoplasma detection kit (Supplier name:Beyotime; Catalogue number: C0301S). RPMI8226 and KMS11 cells stably transfected with NCX1-overexpress lentivirus (established by Shanghai HANBIO Company, China) or NCX1-shRNA lentivirus (established by Shanghai Genechem Company, China) were named RPMI8226-oeNCX1 / KMS11-oeNCX1 or RPMI8226-shNCX1 / KMS11-shNCX1, respectively. The control cell lines named RPMI8226-oeCON / KMS11-oeCON or RPMI8226-shCON / KMS11-shCON with the same empty vectors were developed in the same way.

### Evaluation of fluorescent LC3 puncta

RPMI8226 and KMS11 cells cultured on confocal dishes were transduced with mRFP-GFP-LC3 lentivirus(established by Shanghai HANBIO Company, China) at 10 MOI after different intervention, including CaCl_2_ (Supplier name:Sigma-Aldrich; Catalogue number:10043-52-4)/KB-R7943 (Suppliername:Sigma-Aldrich; Catalogue number:182004-64-4)/BAPTA(Suppliername:Sigma-Aldrich; Catalogue number: 126150-97-8)/CQ(Supplier name: Beyotime; Catalogue number:54-05-7) drug treatment for 48 hours. 24 hours after lentivirus transduction, the cells were observed under a confocal microscope. The number of GFP and mRFP spots was determined by manually counting fluorescent spots in five fields of view with a 100× oil immersion objective.

### Transmission Electron Microscopy (TEM)

MM Cells were collected by centrifugation, and fixed with 2.5% glutaraldehyde at room temperature for 2 hours, then 4 ℃ for overnight, followed by dehydration. Thin sections (50 nm) were cut on an ultramicrotome and stained with uranyl acetate and lead citrate. Images were observed by transmission electron microscopy.

### Colony formation assay

RPMI8226-oeCON, RPMI8226-oeNCX1, KMS11-oeCON and KMS11-oeNCX1 cells were seeded in 6-well plates (400 cells/well) and fed with RPMI 1640 complete medium in the presence or absence of chloroquine (CQ) or BTZ (Supplier name: Velcade) 3 times a week. After 14 days of observation, one colony was defined as more than 40 cells. Cells were then fixed with 3.7% formaldehyde (Supplier name: Meilunbio) for 20 min, followed by staining with crystal violet solution (Supplier name: Meilunbio;Catalogue number:MB4721) for 10 min. The plate was imaged and colonies were counted. Each sample was repeated three times.

### Cell viability analysis

Cell viability was detected by the Cell Counting Kit-8 (CCK-8) (Supplier name: Meilunbio; Catalogue number:MA0218). Briefly, MM cells were planted in triplicate in 96-well plates, then incubated at 37℃ for 24, 48, and 72 hours. Assess cell viability by CCK-8 assay according to the manufacturer's instructions. The cells to be treated include: 1) RPMI8226 and KMS11 cells treated with or without KB-R7943 or BTZ; RPMI8226-shCON, RPMI8226-shNCX1, KMS11-shCON and KMS11-shNCX1 treated with or without BTZ. 2) RPMI8226 and KMS11 cells treated with or without CaCl_2_ or BTZ; 3) RPMI8226 and KMS11 cells treated with or without CaCl_2_, BTZ , KB-R7943 or BAPTA. 4)RPMI8226-oeCON, RPMI8226-oeNCX1, KMS11-oeCON and KMS11-oeNCX1 treated with or without BTZ or CQ.

### Apoptosis and cell cycle analysis

Cell apoptosis was assessed by APC-Annexin V/PI cell apoptosis kit (Supplier name: UElandy;Catalogue number:A6030L). RPMI8226 and KMS11cells were transfected with NCX1-shRNA or added with KB-R7943, followed by BTZ treatment as indicated. Collect the cells by centrifugation and perform Annexin V and PI double staining according to the kit manufacturer's instructions. Annexin V and PI were used to mark apoptotic and dead cells, respectively. Cells were detected and analyzed using the Accuri C6 flow cytometer (BD biosciences).

For cell cycle analysis, RPMI8226 and KMS11cells incubated with or without KB-R7943 or BTZ for 48h, and then washed with PBS, fixed with cold 70% ethanol overnight at 4°C. Next day, cells were centrifuged and resuspended in PBS, and then stained with propidium iodide for 30 minutes under dark conditions at 37°C according to the operating protocol of the cell cycle and apoptosis detection kit (Supplier name: Beyotime;Catalogue number:C1052). Data were acquired by flow cytometry at the wavelength of 488 nm (BD biosciences), and processed and analyzed using FlowJo software.

### Western blot analysis

Treated and untreated cells (RPMI8226 or KMS11) were harvested and total proteins were extracted by lysed in RIPA strong buffer (Supplier name:Meilunbio;Catalogue number:MA0151) with added proteinase inhibitor. Cytoplasmic and nuclear proteins were isolated according to the instructions of the Cytoplasmic and Nuclear Protein Extraction Kit (Supplier name:Beijing TransGen Biotech;Catalogue number:DE201-01). After quantification, protein extracts were separated by 7.5%–15% SDS-PAGE (Supplier name:Shanghai Epizyme Biomedical Technology Co., Ltd;Catalogue number:PG111-114, and transferred to a polyvinylidene difluoride (PVDF) membrane(Supplier name:Merck Millipore Ltd;Catalogue number:ISEQ00010 PORE SIZE.0.2μm). Subsequently, PVDF membranes were sealed with 5% skimmed milk powder in Tris-buffered saline (TBS) containing 0.05% Tween-20 (TBST) for 1-2 hours before incubation with primary antibody overnight at 4 ℃. The next day, membranes were washed 3 times and incubated with horseradish peroxidase-conjugated secondary antibody (Supplier name: Beijing TransGen Biotech; Catalogue number:HS201-01 and HS101-01) at room temperature for 2 hours, followed washed 3 times, and visualized on the ECL detection system (BIO-RAD). Densitometric analyses of blots were performed by using the software ImageJ. Primary antibodies used include: NCX1 (Supplier name:Abcam;Catalogue number: Cat. No. ab177952), ATG7 (Supplier name:ImmunoWay Biotechnology;Catalogue number: Cat. No. Y5670), ATG5 (Supplier name:Boster;Catalogue number: Cat. No.BM4603), P62 (Supplier name: Abclonal;Catalogue number: Cat. No. A11483), LC3B (Supplier name:Abcam;Catalogue number: Cat. No. Ab63817), P100 (Supplier name:ImmunoWay Biotechnology;Catalogue number: Cat. No.YT3093), P52 (Supplier name:ImmunoWay Biotechnology;Catalogue number: Cat. No. YC0200), RelB (Supplier name:ImmunoWay Biotechnology;Catalogue number: Cat. No.YT4045), P105/P50 (Supplier name:ImmunoWay Biotechnology;Catalogue number: Cat. No.YT3101), P-P65 (Supplier name:CST, Cat. No.3033), P65 (Supplier name:CST, Cat. No.8242), β-actin (Supplier name:TransGen Biotecha;Catalogue number: Cat. No. HC201-01), Histone H3 (Supplier name:ImmunoWay Biotechnology;Catalogue number: Cat. No. YM3038).

### Quantitative real-time PCR

Quantitative real-time PCR was used to verify the transfection efficiency of MM cells transfected with NCX1-shRNA or NCX1-overexpression lentivirus. Firstly, the total RNA of MM cells was extracted using TRIzol (Supplier name:Life technologies;Catalogue number:15596026) reagent, and reverse-transcribed using a cDNA reverse transcription kit (Supplier name:Beijing TransGen Biotech;Catalogue number:AT311-02). Each 10ul reaction contained cDNA (1ul), forward (0.5 μl) and reverse primers (0.5 μL) and 2 × SYBR qPCR superMix (8 μL) (Supplier name:Beijing TransGen Biotech;Catalogue number: AS122-01) for amplification. Cycling conditions including 95℃ for 1 min, 40 cycles of 95℃ for 10 s and 60℃ for 60 s. The quantitative expression of the target gene was corrected by β-actin, and the statistical data were analyzed by the 2^−ΔΔCT^ method. Repeat three times for each sample. Primers are constructed by Sangon Biotech, including: β-actin (forward primer: 5′-GGC ATC CAC GAA ACT ACC TT-3′; reverse primer: 5′-CGG ACT CGT CAT ACT CCT GCT-3′); NCX1 (forward primer: 5′-TGT GCA TCT CAG CAA TGT CA- 3′, reverse primer: 5′-TTC CTC GAG CTC CAG ATG TT- 3′).

### Luciferase activity assay

RPMI8226 cells (1x10^4^/well) were seeded into 24-well plates prior to luciferase reporter assay. At 24 hours, cells were transfected with plasmids. The pGL3-based construct containing NCX1 promoter and Renilla luciferase plasmid, as well as NFκB2 overexpression plasmid or a vector control plasmid were co-transfected into cells by Lipofectamine 8000 (Supplier name:Beyond;Catalogue number:C0533). After 48 hours, MM cells were washed with PBS solution and lysed with lysis buffer on ice for 10 minutes to collect proteins. The luciferase and Renilla activities were measured by a dual-luciferase reporter measurement system (Supplier name:YEASEN;Catalogue number:11404ES60), and the firefly luciferase activity was normalized to Renilla activit.

### Myeloma xenograft mouse model

A mouse xenograft myeloma model was used to evaluate NCX1 inhibitor or NCX1-knockdown synergized with BTZ to suppress MM tumors in vivo. 40 male NCG mice (aged 4 weeks) were obtained from the Guangdong Gempharmatech company. Randomly divided into two cohorts, 20 in each cohort. 1×10^7^ KMS-shCON or KMS-shNCX1 cells suspended in 200μl PBS buffer were subcutaneously injected into the right side of the first cohort, and 1×10^7^ RPMI8226 cells were subcutaneously injected into the second cohort. After 10 days, when the tumor diameter reached a size of approximately 0.5 cm, each cohort was randomly divided into 4 groups (5 mice per group). First cohort treated with PBS (control group) or BTZ (10 mg/kg, twice a week). Treatments of the second cohort included the intraperitonial injection of PBS (control group), KB-R7943 (5 mg/kg, 3 times a week), BTZ (10 mg/kg, twice a week) or KB-R7943 plus BTZ. The mice body weight was measured every 7 days, and the tumor was measured with a caliper. The tumor volume (mm^3^) was calculated as π/6×length×width×height. Mice were sacrificed by cervical dislocation after 25 days of treatment. Tumors were fixed with 10% formaldehyde and embedded in paraffin. All mice were housed at the facilities of the Animal Center of Fujian Medical University, and all procedures used in these experiments were approved by the Medicine Institutional Animal Care and Use Committee of Fujian Medical University(number for approval:IACUC FJMU 2023-Y-0492).

### Immunochemistry

Immunohistochemical staining was performed on paraffin-embedded sections (thickness, 5 μm) of human BM tissue and mouse xenograft tumors. Sections were stained using indirect immunoperoxidase method with antibodies NCX1 (1:200) (Supplier name:Abcam;Catalogue number:ab2869), CD138 (1:100) (Supplier name:Immunoway;Catalogue number:YT5610), Ki-67 (1:100) (Supplier name:Immunoway;Catalogue number:YT2467). Images were acquired by microscopic observation. Imagepro Plus measured integrated optical density (IOD) SUM and positive area. Average Density were calculated (IOD SUM)/Area) and presented as mean ± standard deviation (SD).

### Calcium influx assay-Ca^2+^ imaging

Measure the level of [Ca^2+^]_i_ using Fura-4 AM(Beyotime, China), a cell-permeable fluorescent calcium indicator. Incubate MM cell suspension on a polylysine treated cover glass for 24 hours, load 3 uM Fura-4 AM in PSS at 37°C for 30 minutes before measurement, and then washed with PSS for 20 minutes. Install the cover glass in an open perfusion chamber and continuously infuse with PSS. The fluorescence ratio of 340/380 was measured from the region of interest within the cytoplasm. Real time images were captured using a falling fluorescence Nikon Eclipse Ti microscope (x40 objective) and EasyRatioPro software (Photon Technology International). After establishing a stable baseline, the cells were sequentially exposed to PSS with or without CaCl_2_ and KB-R7943. The concentration of [Ca^2+^]_i_ was quantified based on the ratio of 340/380 fluorescence intensity. In each experiment, measure the concentration of [Ca^2+^]_i_ in 10 cells and take the average value.

### Calcium influx assay-flow cytometry

The fluorescence intensity of [Ca^2+^]_i_ was detected by flow cytometry, excited with 488 mm, and the fluorescence signal was collected by the FLI-H fluorescence channel. 1 × 10^4^ cells were collected, and the [Ca^2+^]_i_ concentration was expressed as the mean fluorescence.

### Statistical analysis

All data were statistically analyzed by Graphpad Prism 8.0 software. Data significance was assessed by Student's t-test and analysis of variance test. Overall survival (OS) of MM patients was determined using the Kaplan–Meier method with 95% confidence intervals. *P*<0.05 was considered statistically significant.

## Result

### NCX1 expression is associated with disease prognosis and BTZ sensitivity in human MM

**Fig. 1:**
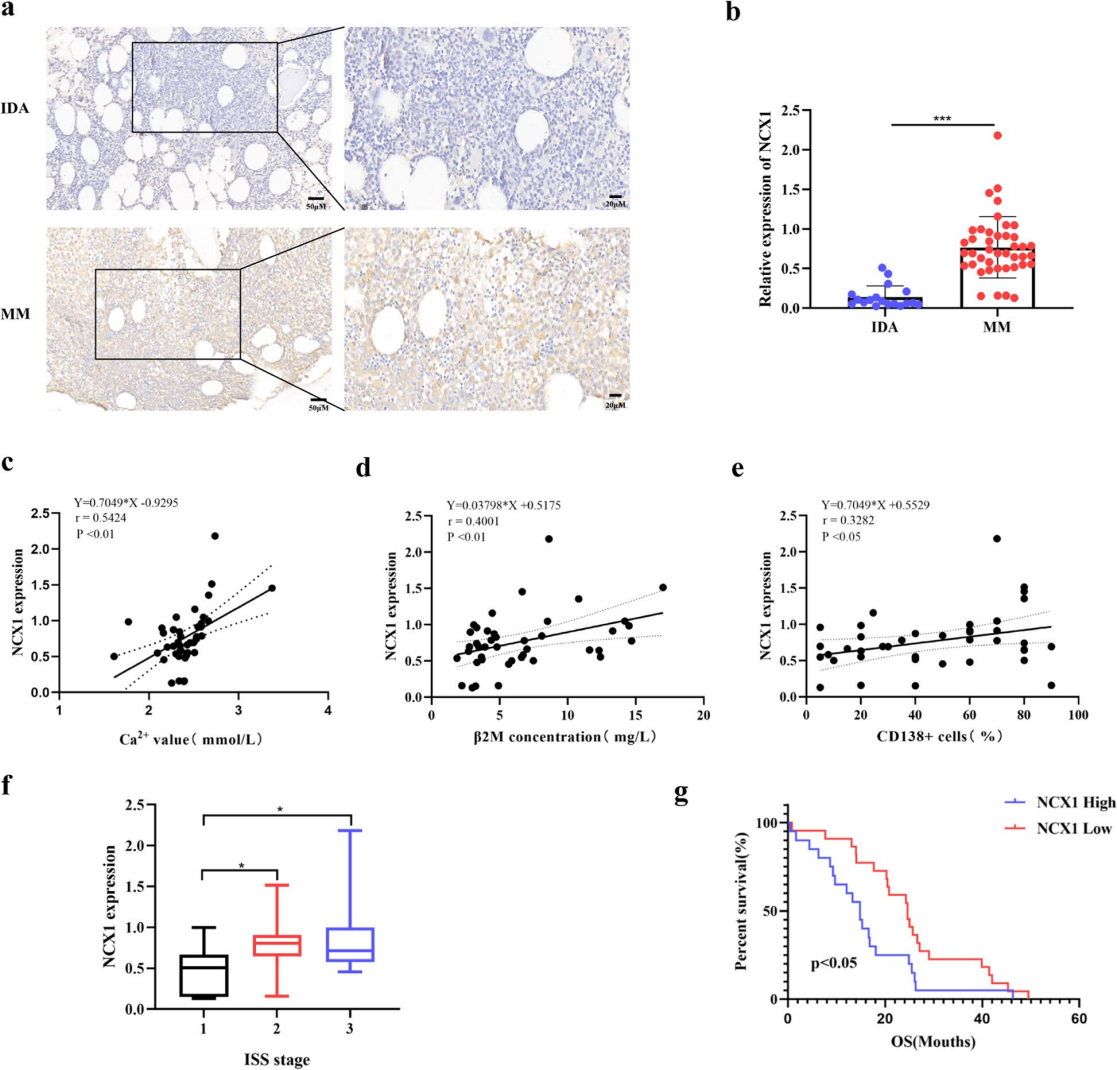
NCX1 expression is associated with disease prognosis and bortezomib sensitivity in human MM. **a** Detection of NCX1 protein expression in bone marrow tissue of MM patients(*n*=42) and iron deficiency anemia (*n*=18) by immunohistochemical staining (Scale bar = 50 μm or 20μm), and summary data (**b**) (***P < 0.001). **c-e** Expression patterns of NCX1 with calcium(**c**), beta-2 microglobulin(**d**) and percentage of bone marrow CD138+ cells(**e**)(*p<0.05, **p<0.01). **f** Correlated NCX1 expression with ISS stage (*p<0.05). **g** The protein expression of NCX1 and the overall survival of MM patients who received bortezomib treatment were analyzed by Kaplan-Meier curve (*p<0.05). The mean value was used as a cutoff point to define low and high NCX1 expression groups

Previously, our group found that NCX1 was highly expressed in myeloma cell lines (RPMI8226, KMS11, U266, MM1S) and human MM BM tissues [15]. To further investigate the impact of NCX1 on MM prognosis and BTZ sensitivity, we proceeded to collect BM tissues from 42 newly diagnosed myeloma patients who had received two or more courses of BTZ treatment. The clinical characteristics and parameters in the 42 newly diagnosed MM patients are summarized in Tables 1 and 2. We examined the protein expression of NCX1 in BM samples from 42 MM patients and 18 IDA by IHC. Consistently, NCX1 expression was significantly higher in MM BM than in corresponding IDA BM samples (Fig. 1a, b).

**Table 1:** Clinical feature of 42 newly diagnosed MM patients from CHINA

Clinical feature	No. of cases(%)
Gender	
Male	21 (50%)
Female	21 (50%)
Age (years)	
<60	15(36%)
≥60	27(64%)
ISS Stage	
Stage I	7(17%)
Stage II	16(38%)
Stage III	19(45%)
M component at diagnosis	
IgG type	18 (43%)
IgA type	11 (26%)
IgM type	0 (0%)
IgD type	2 (5%)
Light chain type	11 (26%)
Kappa type	23 (55%)
Lambda type	19 (45%)

*Abbreviations*: *ISS* International Staging System

**Table 2:** Clinical and pathological characteristics of 42 newly diagnosed MM patients

Clinical parameters	Mean ± SD
Albumin (g/L)	35.71 ± 6.37
Hemoglobin (g/L)	95.13 ± 20.45
Calcium (mmol/L)	2.41 ± 0.27
β2M (mg/L)	6.82 ± 4.12
LDH (U/L)	216.42 ± 108.50
Creatinine (μmol/L)	129.97 ±144.68
IgG (g/L)	25.47 ± 30.45
IgA (g/L)	7.31 ± 17.9
IgM (g/L)	0.30 ± 0.29
Serum kappa (g/L)	26.96 ± 32.59
Serum lambda (g/L)	10.57 ± 21.87
Urine kappa (mg/L)	1219.50 ± 2426.72
Urine lambda (mg/L)	717.82 ± 1799.36

*Abbreviations*: *β2M* Beta-2-microglobulin, *LDH* Lactate dehydrogenase

Next, to explore the clinical and pathological role of NCX1 in MM, we correlated NCX1 expression with clinical parameters in MM patients. We found that the protein expression of NCX1 was positively corrected with serum calcium levels, beta-2-microglobulin (β2M) and the percentage of BM CD138+ cells (Fig. 1c-e). Interestingly, NCX1 expression was increased in BM tissues of MM patients with ISS stage 2 or 3 compared with ISS stage 1 (Fig. 1f). Subsequently, we explored the relationship between the protein expression of NCX1 and the survival of MM patients treated with BTZ by Kaplan-Meier survival curve. As shown in Fig. 1g, MM patients with low NCX1 protein expression had better median overall survival (OS) (24.6 months vs. 14.8 months, *p*<0.05). Taken together, these findings suggest that NCX1 expression was elevated in MM BM tissues and correlated with disease progression in those patients receiving BTZ treatment.

### NCX1 inhibition synergizes with bortezomib anti-MM activity in vitro

**Fig. 2:**
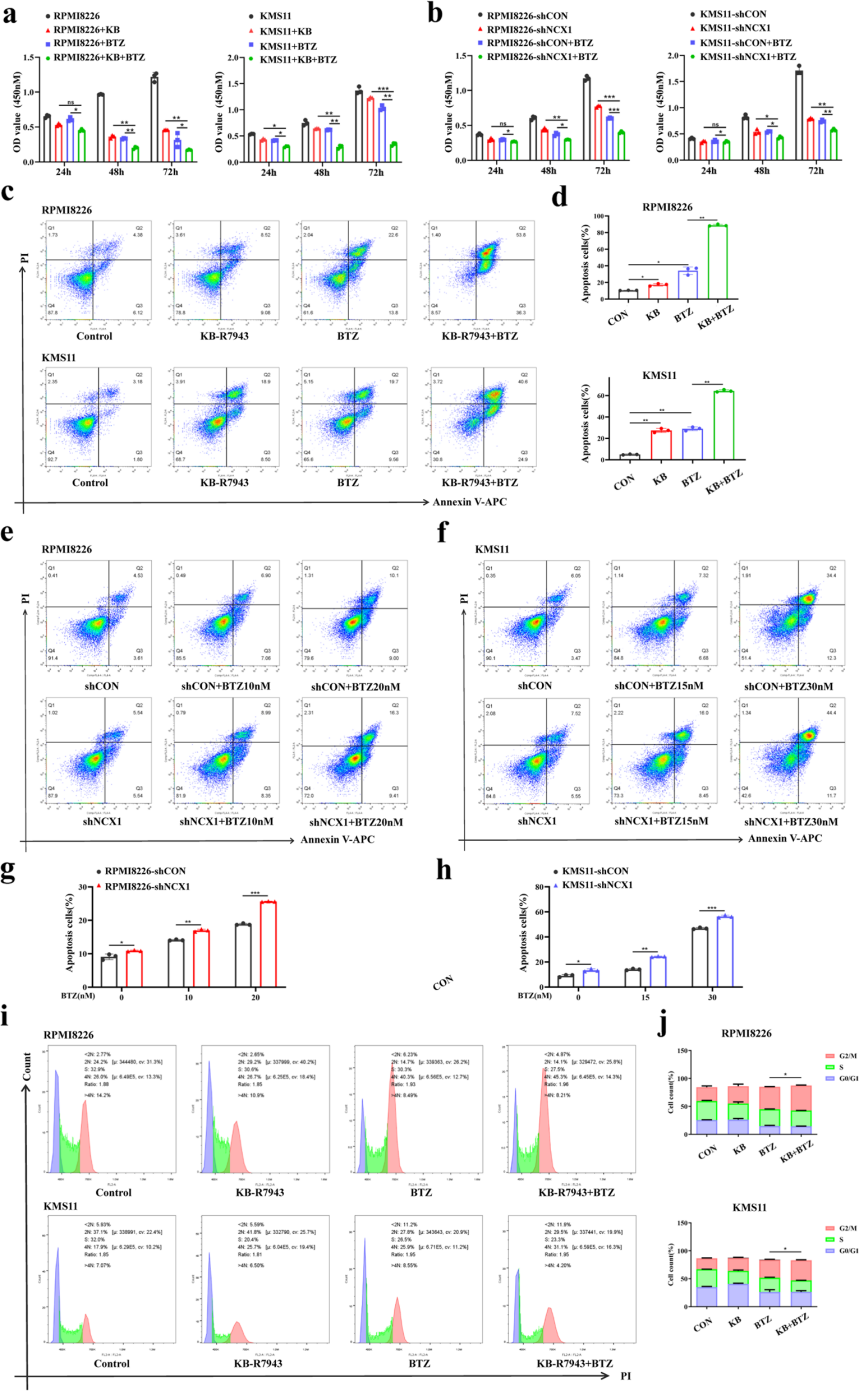
NCX1 inhibition synergizes with bortezomib anti-MM activity in vitro. **a** RPMI8226 and KMS11 cells were exposed to KB-R7943 (20μM) and BTZ (10nM) for 24h,48h and 72h, and cell proliferation was detected by CCK8 assay (*p<0.05, **p<0.01, ***p<0.001, *n*=3). **b** After knockdown NCX1 in RPMI8226 and KMS11, the cells were treated with 10nM BTZ for 24h,48h and 72h,and then detected by CCK-8 cell proliferation assay (*p<0.05, **p<0.01, ***p<0.001, *n*=3). **c** Cell apoptosis was detected by flow cytometry in RPMI8226 and KMS11cell lines treated with 20μM KB-R7943 and 10nM BTZ for 48 h, and summary data(**d**) (*p<0.05, **p< 0.01, *n* = 3). **e-h** Cells apoptosis in NCX1- knockdown of RPMI8226 and KMS11 cells treated with different concentrations of BTZ for 48 h, and summary data (**g**, **h**) (*p<0.05, **p<0.01, ***P < 0.001, *n* = 3). **i**, **j** Cell cycle assay results showed the percentage of cells in G1, S, and G2/M phase in RPMI8226 and KMS11 cells treated with KB-R7943 or/and BTZ for 48h. Histogram summarizes the percentage of cells in G1, S and G2/M stages in three independent experiments (**j**) (*p<0.05, *n* = 3)

In previous study, we have determined that inhibition of NCX1 is able to inhibit MM cell proliferation and induce apoptosis. To further investigate the effect of NCX1 on the sensitivity of MM cells to BTZ, we added the NCX1-specific inhibitor KB-R7943 or knocked down NCX1 in two MM cell lines, RPMI8226 and KMS11 cells, respectively, and exposed them to 10 nM BTZ. The transfection efficiency of knockdown NCX1 in RPMI8226 and KMS11 cells was shown in Additonal 2a. First, CCK8 assay results showed that compared with each single agent alone, a more pronounced inhibition of cell proliferation was observed in RPMI8226 and KMS11 cells treated with KB-R7943 plus BTZ (Fig. 2a). In addition, a similar effect was observed in NCX1 knocking-down combined with BTZ treatment (Fig. 2b), indicating that inhibition of NCX1 synergized with BTZ to suppress MM cell viability. Second, we performed flow cytometry to assess MM cells apoptosis, and the results suggested that inhibition NCX1 using KB-R7943 or knockdown NCX1 enhanced the pro-apoptotic efficacy of BTZ in RPMI8226 and KMS11 cells (Fig. 2c-h). Third, cell cycle results showed that inhibition of NCX1 increased BTZ-induced G2/M arrest and S-interphase shortening in RPMI8226 and KMS11 cells (Fig. 2i-j). These data indicated that NCX1 inhibition can synergize with BTZ to enhance anti-MM cells activity.

### High extracellular calcium([Ca^2+^]_o_) promotes NCX1 expression and autophagic flux in MM cells

**Fig. 3:**
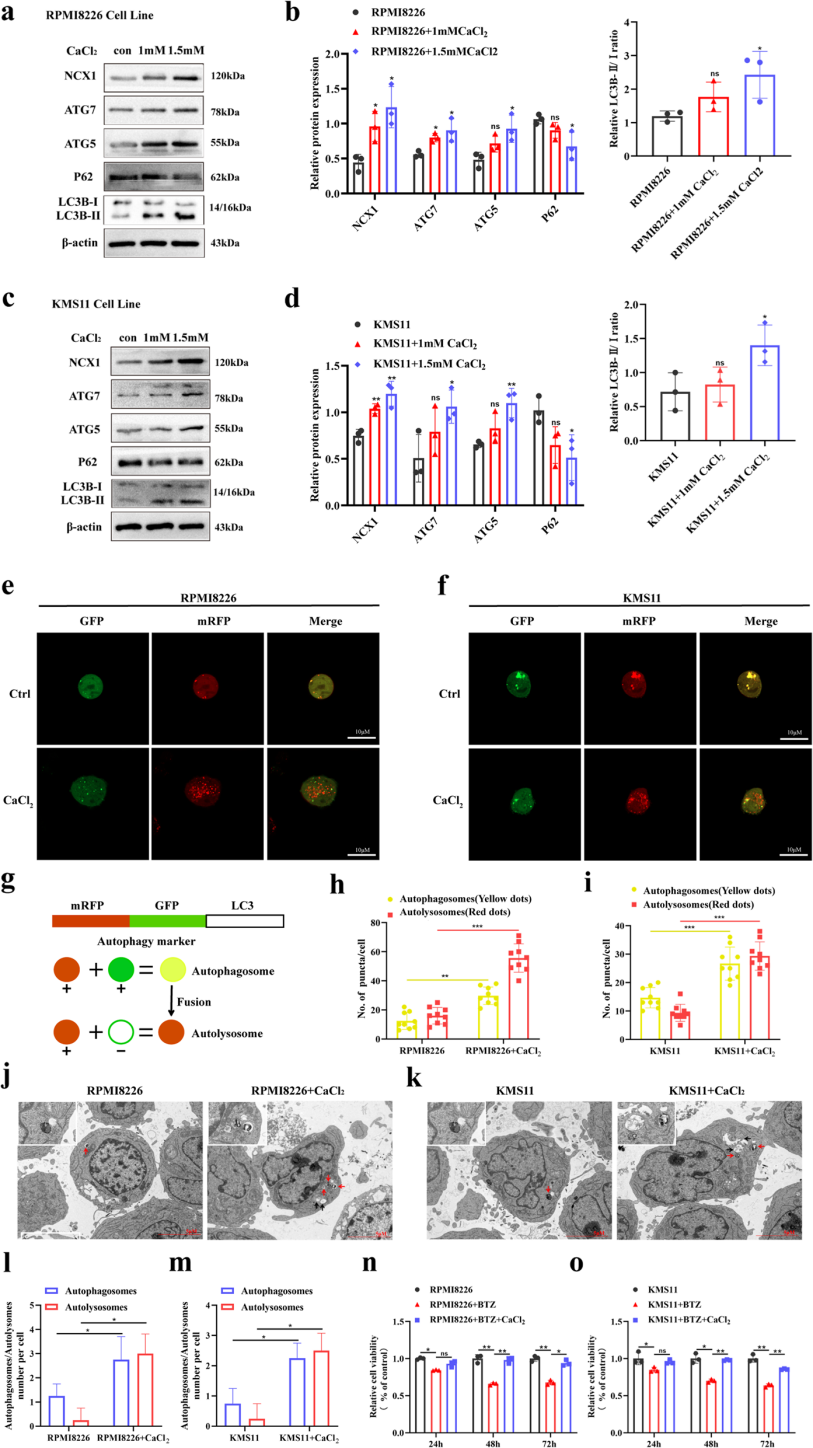
High extracellular calcium([Ca^2+^]_o_) promotes autophagic flux. **a,c** RPMI8226 cells and KMS11cells were treated with or without CaCl_2_ (1mM and 1.5mM) for 48h, followed by Western Blot to determin NCX1, ATG7, ATG5, P62 and LC3B-II/I levels under each condition, and summary data (**b**, **d**) (*p<0.05, **p<0.01, *n* = 3). **e**, **f** The confocal microscopy images of RPMI8226 and KMS11 cells showed more yellow (autophagosomes) and red dots (autolysosomes) after treating with CaCl_2_ (1.5mM) for 48h. Scale bar is 10µm. **g** The autophagosome marker protein LC3 was tandem labeled with RFP and GFP, and the autophage appeared yellow dots, while autolysosomes appeared red dots. **h**, **i** Histogram of the number of yellow dots and red dots. (**p<0.01, ***p<0.001, *n* = 9). **j**, **k** TEM analysis of autophagosomes (red arrow) and autolysosomes (black arrow) in RPMI8226 and KMS11 cells treated with or without CaCl_2_, and summary data (**l**, **m**) (*p<0.05, *n* = 4). Scale bar: 5 or 1μm. **n**, **o** RPMI8226 and KMS11cells were treated with BTZ (10nM) or/and CaCl_2_(1.5mM) for 24h, 48 h, 72h. The viability was detected by CCK8 assay (*p<0.05, **p<0.01, *n* = 3)

It is well known that cellular calcium homeostasis and autophagy play important roles in BTZ sensitivity of MM cells [26, 27]. Our previous work detected [Ca^2+^]_i_ by flow cytometry and showed that [Ca^2+^]_o_ activated NCX1, promoted extracellular calcium influx [21], which we further demonstrated using calcium ion imaging system (Additional 1). Here, we would like to further investigate the potential effects of high calcium microenvironment activating NCX1 on autophagy and BTZ sensitivity in MM cells. As shown in Fig. 3a-d, under basal conditions, [Ca^2+^]_o_ increased NCX1 expression and induced a significant increase in autophagy marker proteins (ATG7, ATG5, and LC3B-II), and caused an increase in the clearance of p62, an autophagy cargo receptor protein [28], suggesting enhanced autophagy in RPMI8226 and KMS11 cells. Next, we transduced MM cells with mRFP-GFP-LC3 tandem fluorescent protein lentivirus to evaluated the extent of autophagosome and autolysosome formation. The numbers of autophagosome (yellow dots) and autolysosome (red dots) per cell were both significantly increased after incubation in the medium with higher calcium concentration, and more free red dots than yellow dots were seen (Fig. 3e-i), suggesting that [Ca^2+^]_o_ increases autophagic flux. Subsequently, transmission electron microscopy (TEM) showed that autophagosomes and autolysosomes were increased in RPMI8226 and KMS11 cells treated with CaCl_2_ (Fig. 3j-m). Moreover, as shown in Fig. 3k and l, the increased [Ca^2+^]_o_ inhibited the sensitivity of BTZ in MM cells.

### Inhibition of NCX1 reverses the effect of higher [Ca^2+^]_o_ increasing autophagic flux in MM cells

**Fig. 4:**
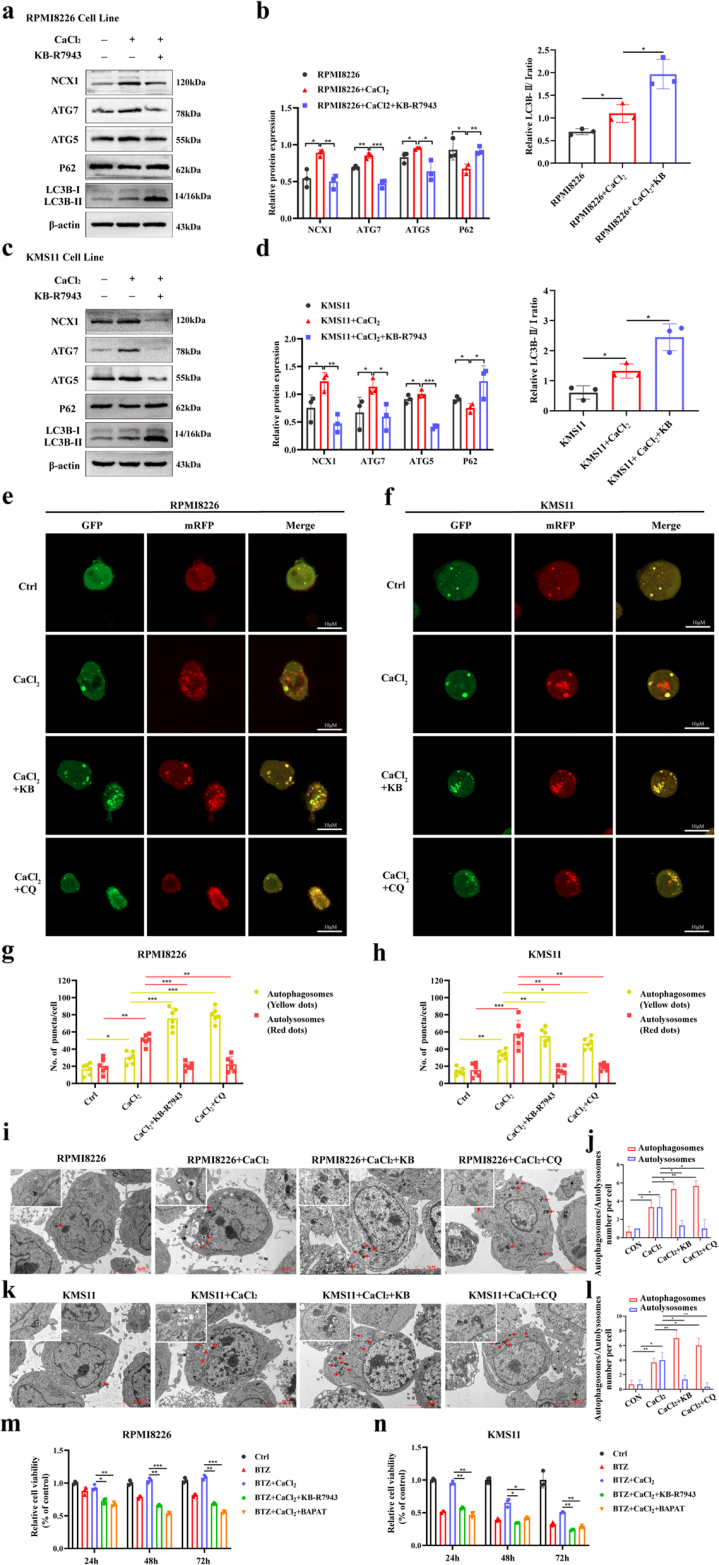
Inhibition of NCX1 reverses the high [Ca^2+^]_o_ induced increase in autophagic flux in MM cells. **a**, **b** RPMI8226 and KMS11 cells were treated with 1.5mM CaCl_2_ in combination with or without KB-R7943 for 48h. NCX1 protein and autophagic marker proteins (ATG7, ATG5, P62 and LC3B-II/I) were detected by western blot , and summary data (**b**, **d**) (*p<0.05, **p<0.01, ***p<0.001, *n* = 3). **e**, **f** Representative images of confocal microscopy in RPMI8226 and KMS11 cells transduced with mRFP-GFP-LC3 lentivirus and treated with 1.5mM CaCl_2_ in combination with or without KB-R7943/CQ for 48h. Scale bar is 10µm, and Histogram of the number of yellow dots (autophagosomes) and red dots (autolysosomes)(**g**, **h**) (*p<0.05, **p<0.01, ***p<0.001, *n* = 6). **i**, **k** TEM analysis of autophagosomes (red arrow) and autolysosomes (black arrow) in RPMI8226 and KMS11 cells treated with CaCl_2_ in combination with or without KB-R7943/CQ, and summary data (**j**, **l**) (*p<0.05, **p<0.01, *n* = 3). Scale bar: 5 or 1μm. **m**, **n** RPMI8226 and KMS11cells were treated with 10nM BTZ in combination with 1.5mM CaCl_2_ with or without KB-7943/BAPTA for 24h, 48 h, 72h. The viability of MM cells was detected by CCK8 assay (*p<0.05, **p<0.01, ***p<0.001, *n* = 3)

To further confirm the role of NCX1 in autophagy, we inhibited the expression of NCX1 using KB-R7943, and observed whether NCX1 inhibition can reverse the increase of autophagic flux induced by [Ca^2+^]_o_. As shown in Fig. 4a and c, KB-R7943 reversed the increase in autophagic marker proteins (ATG7, ATG5, and LC3B-II) expression and p62 clearance induced by [Ca^2+^]_o_. Then, to verify whether autophagic marker proteins reversed by KB-R7943 were due to decreased autophagic flux, we treated MM cells with CaCl_2_ and KB-R7943 or chloroquine (CQ). CQ is a late-stage autophagy inhibitor that prevents autophagosome-lysosome fusion [29]. We found that KB-R7943 or CQ can reverse the increased of autophagic flux induced by CaCl_2_ (Fig. 4e-h). Further confirmation of autophagic flux inhibition was obtained by TEM. NCX1 inhibition or autophagy inhibitor markedly elevated the number of autophagosomes, and reversed the autolysosomes increased by CaCl_2_ (Fig. 4i-l).

Of note, BAPTA, an intracellular calcium chelator [30], also showed inhibitory effects on increased autophagy marker proteins, autophagic flux and autophagosomes/autolysosomes induced by CaCl_2_, suggesting that NCX1 regulates autophagy by interfering with calcium homeostasis (Additional 3).

Besides, we also confirmed that CaCl_2_-reduced BTZ sensitivity in MM cells could be reversed by KB-R7943 or BAPTA (Fig. 4m, n). These results demonstrate that NCX1 appears to regulate autophagy and BTZ sensitivity by disturbing calcium homeostasis.

### Inhibition of autophagy sensitizes high NCX1 MM cells to BTZ

**Fig. 5:**
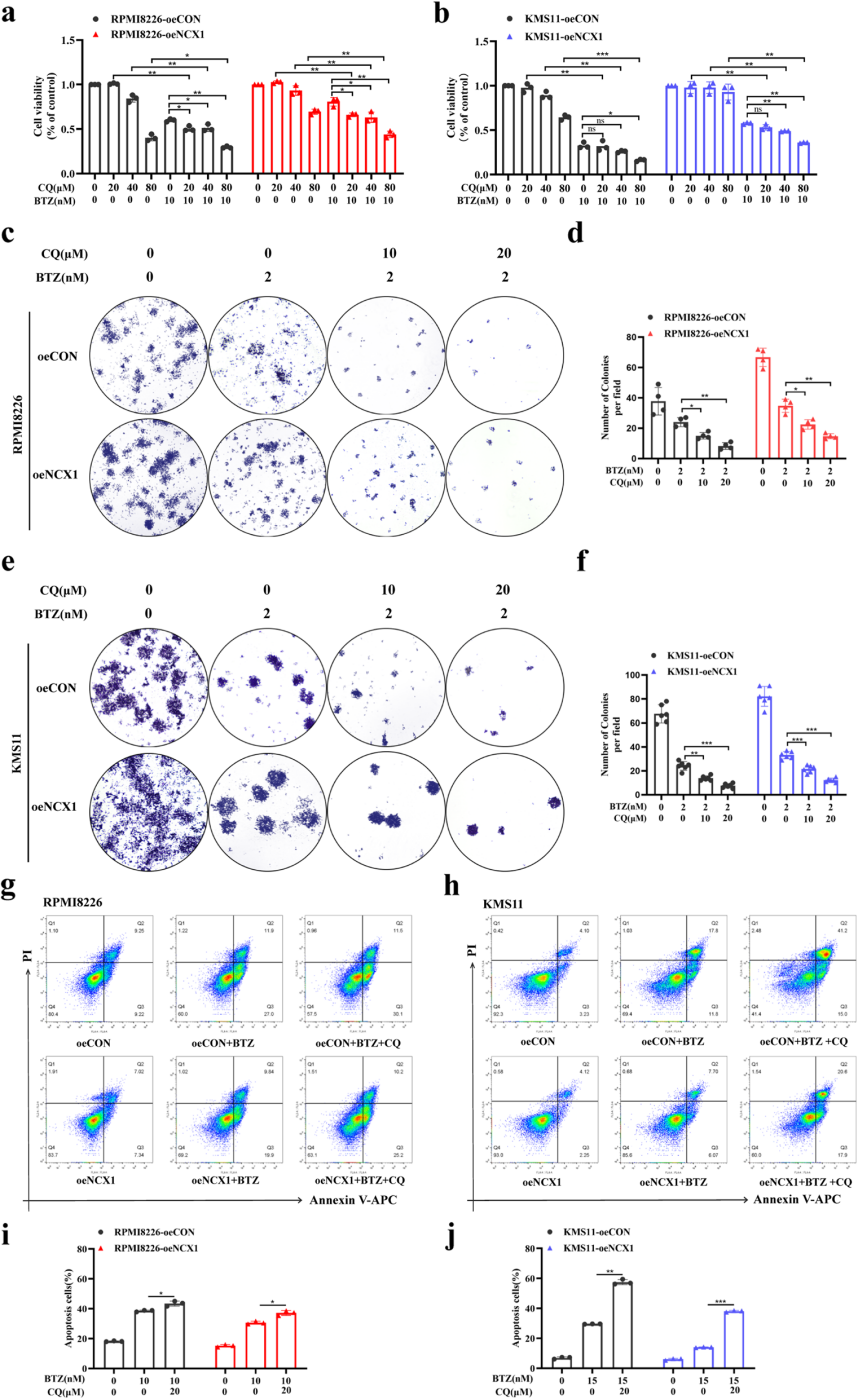
Inhibition of autophagy sensitizes high NCX1 MM cells to BTZ. **a**, **b** RPMI8226-oeCON, RPMI8226-oeNCX1, KMS11-oeCON and KMS11-oeNCX1 cells treated with different concentrations of CQ (0, 20μM, 40μM, 80μM) in combination with or without BTZ(10nM) for 48h. Cell viability was detected by CCK8 assay (*p<0.05, **p<0.01, ***p<0.001, *n*=3). **c,e** Representative images of clonogenic analysis in RPMI8226-oeCON, RPMI8226-oeNCX1, KMS11-oeCON and KMS11-oeNCX1 cells treated with BTZ (2nM) in combination with or not CQ (0, 10μM, 20μM) for 7 days, and summary data (**d**, **f**) (*p<0.05, **p<0.01, ***p<0.001, *n*=4 or *n*=6). **g**, **h** Assessment of cell apoptosis by using Annexin V-APC/PI double staining in RPMI8226-oeCON, RPMI8226-oeNCX1, KMS11-oeCON and KMS11-oeNCX1 cells after treatment with 10nM or 15nM BTZ with or without 20μM CQ for 48h, and summary data (**i**, **j**) (*p<0.05, **p<0.01, ***p<0.001, *n*=3)

Previous studies have reported that targeted inhibition of autophagy is an effective strategy to increase BTZ sensitivity in MM [31, 32]. To further explore the correlation between NCX1 and autophagy in the sensitivity of MM to BTZ, we overexpressed NCX1 in RPMI8226 and KMS11 cells by lentivirus (Additional 2b). Then, MM cells overexpressing NCX1 were treated with BTZ with or without autophagy inhibitor, CQ, for 48 hours. Contrary to the above results that inhibition of NCX1 increased BTZ sensitivity in MM cells (Fig. 2), overexpression of NCX1(oeNCX1) decreased the sensitivity of MM cells to BTZ. However, compared with oeNCX1 MM cells treated with BTZ or CQ alone, the combination of BTZ and CQ significantly increased the inhibition of cell viability in a concentration dependent manner (Fig. 5a, b). Meanwhile, colony formation assay was performed to determine the synergistic effect of BTZ and CQ on inhibiting the proliferation of oeNCX1 MM cells. As shown in Fig. 5c, e, compared with BTZ alone, CQ combined with BTZ showed a more obvious inhibitory effect on colony formation in oeNCX1 MM cells. In addition, the synergistic effect of BTZ and CQ was also confirmed on promoting apoptosis of oeNCX1 RPMI8226 or KMS11 cells. As shown in Fig. 5g, h, compared with oeNCX1 RPMI8226 or KMS11 cells treated with BTZ alone, increased apoptosis in those cells treated with the combination of BTZ and CQ. These outcomes displayed that autophagy inhibition reversed the BTZ-resistant effect of oeNCX1 MM cells.

### NCX1 induces autophagy through non-classic NFκB signaling pathway in MM cells

**Fig. 6:**
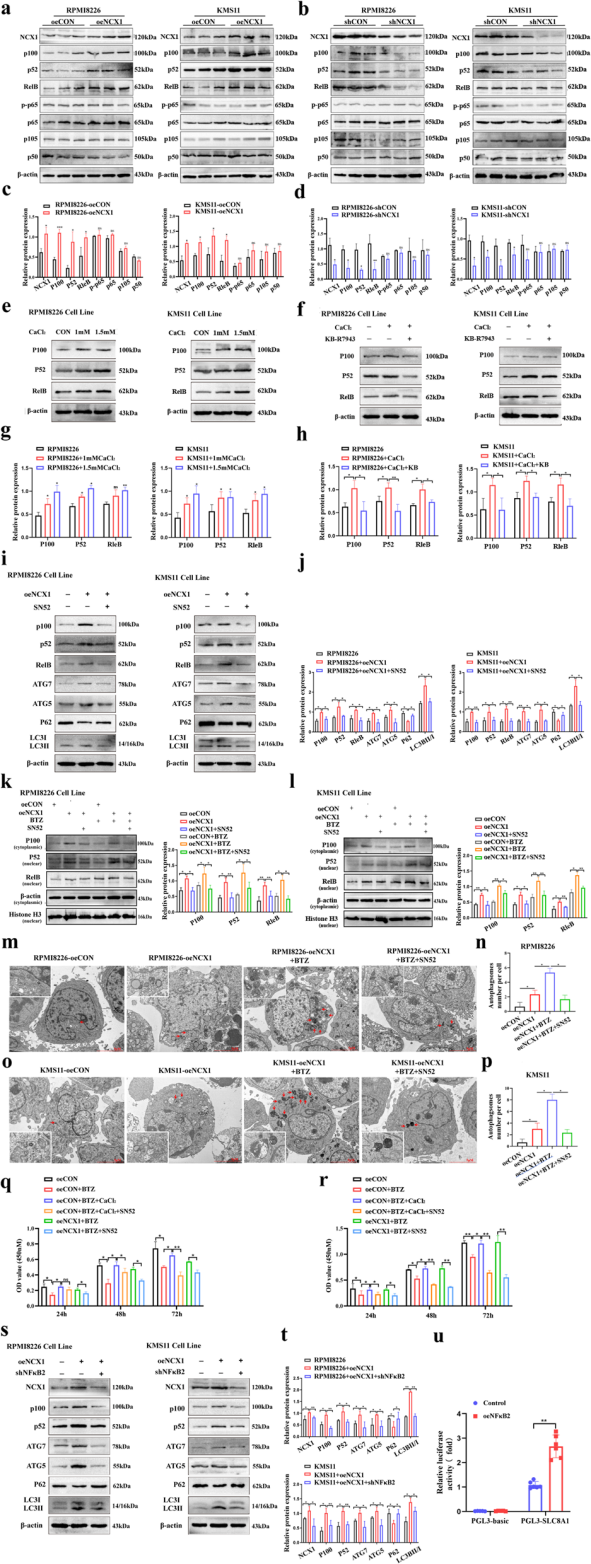
NCX1/Ca^2+^ induces autophagy through non-classic NFκB signaling pathway in MM cells. **a**, **b** Western blotting detection of classical NFκB2 signaling pathway-related proteins(p-p65, p65, p105, p50) and non-canonical NFκB pathway-related proteins (p100, p52, RelB) in NCX1-knockdown or NCX1-overexpression RPMI8226 and KMS11cells, and summary data (**c**, **d**) (*p<0.05, **p<0.01, *n*=3). **e**, **f** KB-R7943 reversed the expression of non-canonical NFκB-associated proteins enhanced by CaCl_2_, and summary data (**g**, **h**) (*p<0.05, **p<0.01, *n* = 3). **i** SN52 abrogated the increasing of p100, p52 and autophagy related proteins expression induced by NCX1-ovexpression in MM cells, and summary data (**j**) (*p<0.05, **p<0.01, *n* = 3). **k**, **l** oeCON and oeNCX1 RPMI8226 and KMS11 cells were exposed to BTZ/SN52 or not. Nuclear protein and cytoplasmic extraction were subjected to western blot using anti-p100, -p52, -RelB and Histone H3 antibodies, and summary data (*p<0.05, **p<0.01, *n*=3). **m**, **o** TEM analysis of autophagosomes (red arrow) in RPMI8226 and KMS11 cells transfected with NCX1-overexpressing lentivirus in combination with or without BTZ/SN52, and summary data (**n**, **p**) (*p<0.05, *n* = 3). Scale bar: 5 or 1μm. **q**, **r** RPMI8226-oeCON, RPMI8226-oeNCX1, KMS11-oeCON and KMS11-oeNCX1 cells were treated with BTZ in combination with CaCl_2_ or SN52. The viability of MM cells was detected by CCK8 assay (*p<0.05, **p<0.01, *n* = 3). **s** Knockdown of NFκB2 abrogated the effects of NCX1 on p100, p52 and autophagy related proteins, and summary data (**t**) (*p<0.05, **p<0.01, *n* = 3). **u** Luciferase reporter assays of the transduced RPMI8226-oeNFκB2 cell transfected with reporter plasmids containing the NCX1 promoter. Transduced RPMI8226 cell transfected with a blank pGL3 plasmid used as a negative control (**p<0.01, *n*=5)

Recent evidence highlighted NFκB-induced autophagy has a tumorigenic effect in most human cancers [33]. Considering that NFκB is the key target of BTZ [34], we proposed to determine whether NFκB can affect NCX1 induced-autophagy activation in MM cells. To explore this, we first investigated the effects on canonical and non-canonical NFκB pathways by overexpressing and knocking down NCX1 in MM cell lines. We found that overexpression of NCX1 significantly increased the expression of non-canonical NFκB-associated proteins (P100, P52, and RelB), but did not affect the canonical NFκB-associated proteins (P105, P50, P-P65, and P65) (Fig. 6a, c). In contrast, knocking down of NCX1 in RPMI8226 and KMS11 cell lines significantly reduced the expression of non-canonical NFκB-related proteins, but also had no effect on canonical NFκB-related proteins (Fig. 6b, d). In addition, we found that CaCl_2_ promoted the expression of non-canonical NFκB-associated proteins, which can be reversed by NCX1 specific inhibitor KB-R7943 (Fig. 6e-h).

To determine whether non-canonical NFκB mediates NCX1-enhanced autophagy, we added NFκB pathway inhibitor SN52 in NCX1-overexpression MM cells. Then, western blot was performed to detect the expression of P100, P52, RleB, ATG7, ATG5, P62 and LC3B-II/I. P100, P52 and RelB proteins were significantly reduced after using SN52 in MM cells. As expected, ATG7, ATG5, and LC3B-II/I were also decreased and the autophagy substrate P62 was increased in NCX1-overexpression MM cells following the addition of SN52 (Fig. 6i, j). Moreover, as revealed in Fig. 6k and l, SN52 attenuated NCX1 and BTZ- induced P52 and RelB nuclear translocation in MM cells. Consistently, TEM results showed that SN52 could reverse the effect of NCX1 /BTZ on increasing the number of autophagic bodies (Fig. 6m-p). In addition, by detecting the viability of MM cells, we found that SN52 reversed the inhibitory effect of NCX1/Ca^2+^ on BTZ sensitivity (Fig. 6q, r).

NFκB2 encodes p100/p52 protein, we constructed NFκB2-shRNA lentiviral system for targeted inhibition in NCX1-overexpression MM cells. Besides knocking down p100/p52 levels, sh-NFκB2 also impaired NCX1-induced autophagy activation (Fig. 6s, t). Of note, knocking down NFκB2 reduced the expressions of NCX1 in MM cells (Fig. 6s, t). We next assessed whether NFκB2 activation promoted the transcription of NCX1. Sequence analysis by JASPAR predicted that NFκB2 has binding sites at the NCX1 promoter. Importantly, this was confirmed by the luciferase reporter assay (Fig. 6u), suggesting the formation of positive feedback loop between NCX1 and activation of the non-canonical NFκB signaling pathway. These results collectively demonstrate the critical role of non-canonical NFκB signaling pathway in NCX1-regulated autophagy and BTZ sensitivity.

### NCX1 inhibition sensitizes MM cells to bortezomib in vivo

**Fig. 7:**
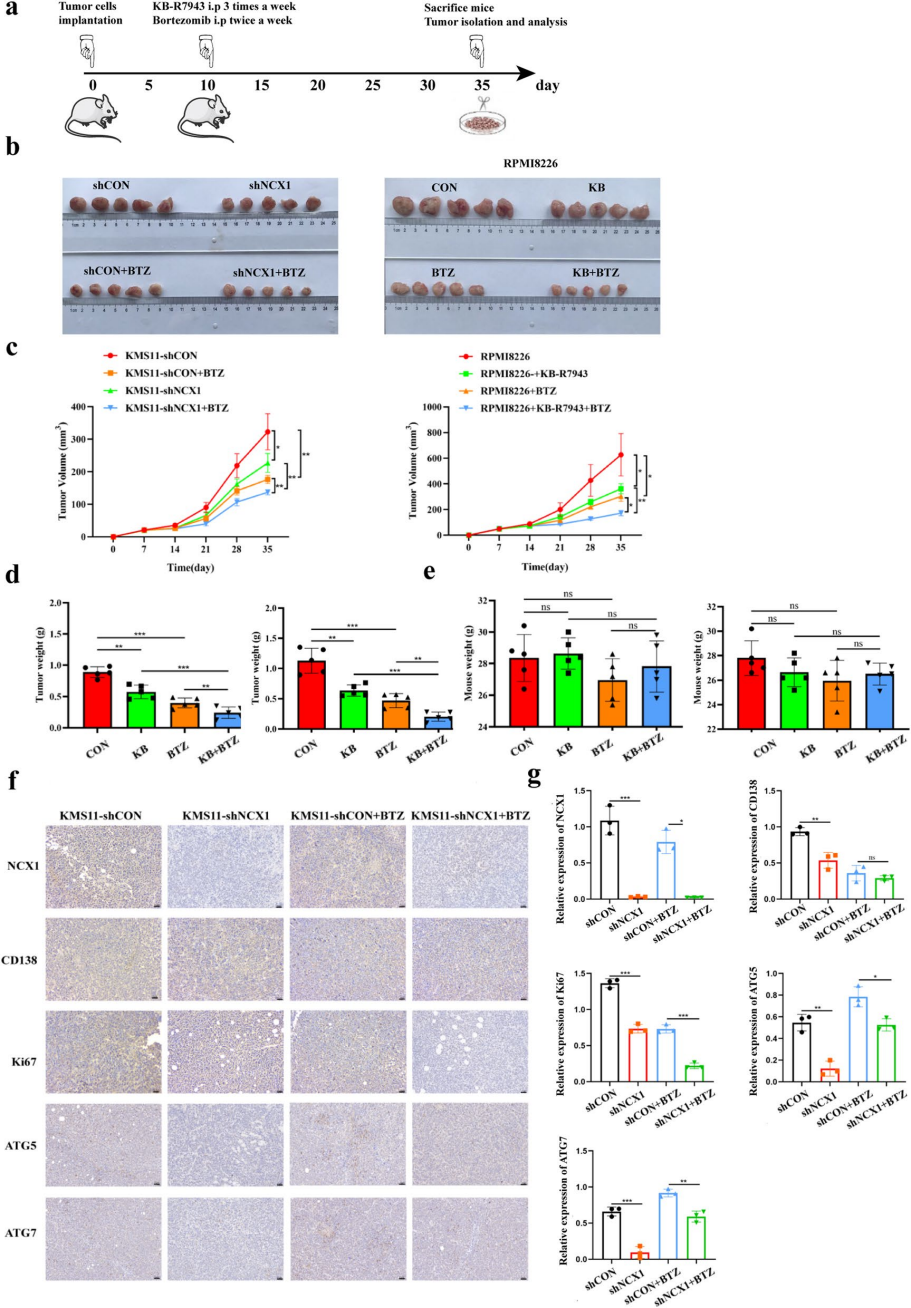
NCX1 inhibition sensitizes MM cells to bortezomib in xenograft mouse models. **a** RPMI8226 and KMS11 cells(1×10^7^) were subcutaneously injected into the right groin of NCG mice, and 10 days later were treated with intraperitoneal bortezomib injections(0.5 mg/kg), twice a week, and intraperitoneal KB-R7943 injections (5 mg/kg) three times a week. On day 35 following MM cell inoculation, mice were sacrificed and tumors were assessed for MM burden. **b** Photographic images of resected tumor from all the mice in each group. **c** Changes in tumor volume. Tumor diameters were measured with calipers once a week (5 mice per group), and tumor volumes were estimated using the following formula v=π/6 * L * W * H, where "L", "W" and "H" are the longest diameter, shortest diameter and height of the tumor respectively. Data are presented as the mean±SD from 5 mice (*p<0.05, **p<0.01, *n*=5). **d** Mean tumor weights at day 35 after inoculation MM cells (**p<0.01, ***p<0.001, *n*=5). **e** Mean body weights at day 35 after inoculation MM cells. **f** Representative images of immunohistochemical staining for NCX1, CD138, Ki67, ATG5 and ATG7 in the subcutaneous tumours, and summary data (g) (*p<0.05, **p<0.01,***p<0.001, *n*=3). The magnifications are 20x. Scale bar:50μm

Lastly, we investigated the effectiveness of KB-R7943 or NCX1 knockdown in combination with BTZ using a MM xenograft NCG mouse model. In this model, NCG mice were divided into two groups (20 in each group), one group was subcutaneously injected with KMS11-shCON and KMS11-shNCX1 cells, and the other group was injected with RPMI8226 cells. Subsequently, KB-R7943, BTZ or their combination were injected intraperitoneally when tumors were palpable subcutaneously (at day 10 after MM cell injection) (Fig. 7a). As demonstrated in Fig. 7b, c, the combination of NCX1-knockdown or KB-R7943 with BTZ caused a greater reduction in tumor growth than either single treatment alone. Tumor growth patterns in mice showed that inhibition of NCX1 and BTZ treatment effectively weakened tumor growth (Fig. 7c, d). Notably, no significant changes in body weight were observed during the treatment period(Fig. 7e), indicating no evidence of toxicity caused by the applied of NCX1-knockdown, KB-R7943 or BTZ and their combination. Moreover, we determined the expression of NCX1, CD138, Ki67, ATG5 and ATG7 in tumor sections by immunohistochemical staining. We observed that combination therapy significantly suppressed the expression of Ki67 compared with single treatment, and knocking down NCX1 not only inhibited the expression of autophagy related proteins ATG5 and ATG7, but also reversed the increased expression of ATG5 and ATG7 induced by BTZ (Fig. 7f, g). Overall, these results recapitulate the in vivo observations, suggesting that targeted inhibition of NCX1 may enhance the anti-MM activity of BTZ in vivo by inhibiting autophagy..

## Discussion

**Fig. 8:**
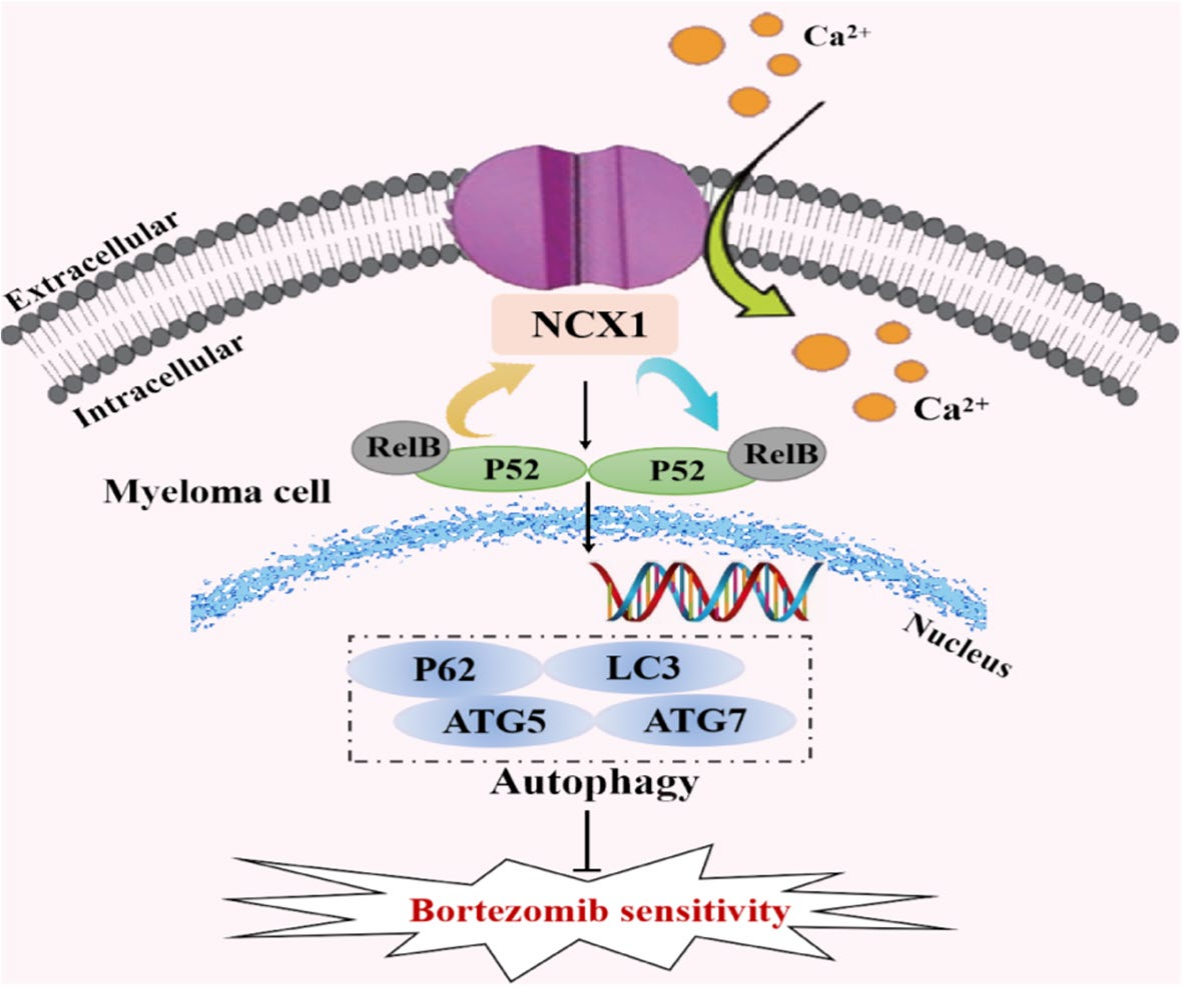
The model of our working

Intensive research has focused on understanding the mechanisms involved in BTZ resistance. Resistance mechanisms discussed in the current mainly include cellular drug pumps, mutations of proteasome and reductions of unfolded protein response (UPR), as well as activation of autophagy level [35]. BTZ exerts its anti-tumor effect by inhibiting protein degradation, while increased autophagy triggers another mode of protein degradation. Autophagy regulates homeostasis by clearing and recycling damaged or useless cellular proteins and organelles [36], promotes tumor cell survival, and inhibits the anti-myeloma efficacy of PI [37, 38]. In this study, we investigated the relationship between NCX1/Ca^2+^ and BTZ sensitivity in MM cells and the underlying molecular mechanisms, with focus on the autophagic process and cell viability. Our results demonstrated for the first time that NCX1 inhibition enhances the chemosensitivity of BTZ in MM. The findings can be summarized as follows: 1) High NCX1 expression in MM was positively correlated with serum calcium, β2M, whereas low NCX1 expression had better OS in MM patients treated with BTZ. 2) Inhibition of NCX1 expression in MM cell lines synergized with BTZ to suppress MM cell viability and trigger apoptosis. 3) Inhibition of NCX1 reversed the autophagic flux and BTZ resistance induced by the high extracellular calcium microenvironment. 4) The inhibitory effect of NCX1/Ca^2+^ on BTZ sensitivity associated with increased autophagy through activating the non-canonical NFκB signaling pathway. 5) NCX1-mediated extracellular calcium influx activated the non-canonical NFκB signaling pathway, while NFκB2 activation promoted NCX1 transcription levels, thus forming a positive feedback loop between NCX1/Ca^2+^ and activation of the non-canonical NFκB signaling pathway. A working model is schematically showed in Fig. 8.

The function of autophagy is controversial in cancer; likely this could be a cytoprotective or cytotoxic mechanism. Recent efforts have focused on targeting cytoprotective autophagy inhibition as a therapeutic strategy to sensitize cells to chemotherapy [39]. Myeloma cells require a specific level of autophagy to survive [40], and inhibition of autophagy induces MM cell death and increases BTZ drug sensitivity [41]. Data from domestic research groups have shown that reducing autophagic flux could overcome BTZ resistance in MM [27, 42]. A clinical pilot study concluded that autophagy inhibitors synergized with BTZ anti-myeloma activity [9]. Consistent with previous work, our study identified that inhibition of autophagy sensitizes high NCX1 MM cells to BTZ. Furthermore, NCX1 inhibition attenuated high Ca^2+^-induced activation of cytoprotective autophagy, thereby increasing BTZ sensitivity. To our knowledge, only a few reports have shown expression of NCX1 in several cancer cells [43–45]. The function of NCX1 in cancer cell development or chemosensitivity remains unknown. In our study, w**e** found that inhibiting NCX1 in MM cells had no obvious effect on [Ca^2+^]_i_ (Additional 1c and d), which maybe due to the fact that intracellular and extracellular calcium was in a state of balance under physiological conditions, and changing the NCX1 channel alone would not affect [Ca^2+^]_i_. However, in the high-calcium microenvironment of MM, [Ca^2+^]_o_ activated the reverse transport mode of NCX1, causing Ca^2+^ influx from the outside (Additional 1a,b and e). Ca^2+^ entered MM cells and acted as a second messenger to activate the NFκB2 signaling pathway and affected autophagy-related proteins (Fig. 6e-h). At the transcription level, NFκB2 promoted the expression of NCX1(Fig. 6u), indicating that in the high Ca^2+^ environment of MM, [Ca^2+^]_o_ was transported into cells through NCX1 channel and promoted NCX1 expression through NFκB2. Our findings highlighted the importance of NCX1/Ca^2+^ signaling in autophagy and suggested the potential of targeting the axis to improve BTZ sensitivity in MM.

Multiple signaling pathways have been described to be involved in autophagy in MM, such as NFκB [46], P38 [47], ERK and mTOR [48]. Our previous research results showed that NCX1 expression had no effect on ERK/pERK and P38/pP38 [21]. It has been reported that NFκB plays an important role in the survival of various B-cell tumors, especially MM [49], and NFκB is a key target of BTZ. Therefore, in this study, we mainly focused on whether NCX1 regulates autophagy and affects BTZ sensitivity through the NFκB signaling pathway. NFκB is a family of transcription factors that includes p65 (RelA), RelB, c-Rel, p50 (NFκB1), and p52 (NFκB2) [50]. It has also been shown that 15-20% of MM involve NFκB pathway mutations [51], which lead to activation of canonical and non-canonical NFκB pathways [52]. Accumulating evidence indicated aberrant non-canonical signaling in cancer [53, 54]. Therefore, identifying the mechanism of downregulation of the non-canonical NFκB signaling pathway was critical for developing a new therapeutic strategie to selective blockade this pathway. In previous report, it had been shown that calcium ion transporters were also involved in the regulation of NFκB activity and function in lymphocytes [55]. In this study, we found that NCX1/Ca^2+^ mainly activated the non-canonical NFκB pathway in MM cells, and inhibition of NCX1 attenuated the Ca^2+^-induced non-canonical NFκB- activition. Importantly, we showed for the first time that the enforced expression of NCX1 combined with BTZ induced the NFκB2 nuclear translocation and autophagy activition in MM cells and that these effects could be reversed by SN52 (Fig. 6). Moreover, SN52 sensitized high NCX1/Ca^2+^ MM cells to BTZ (Fig. 5), which indicating that NCX1 regulated autophagy and BTZ sensitivity through non-classical NFκB pathway in MM.

Our findings have great clinical implications. The proteasome inhibitor BTZ is the cornerstone of anti-MM therapy. However, with the increasing clinical use of BTZ, drug resistance and side effects seriously affect the clinical efficacy and prognosis of MM [56]. Therefore, the search for target which increase BTZ sensitivity are important research topics [57]. In this study, we determined the role of NCX1 in the response of BTZ sensitivity in MM cells. In vitro, inhibition of NCX1 synergized with BTZ to increase the cytotoxic effect on MM cells (Fig. 2). In vivo, NCX1-knockdown combined with BTZ treatment produced superior antitumor effects in a subcutaneous xenograft model of MM mouse compared to either single treatment (Fig. 7). Mechanistically, NCX1/Ca^2+^ was able to activate the non-canonical NFκB pathway, and NFκB2 also regulated the expression of NCX1/Ca^2+^, forming a positive feedback, which subsequently promotes autophagy and the inhibition of BTZ sensitivity in MM cells.

In conclusion, we demonstrated for the first time that low NCX1 enhances the anti-MM activity of BTZ both in vitro and in vivo, which provided a new marker to overcome BTZ resistance and improve MM patient prognosis and survival.

### Supplementary Information

**Supplementary Material 1.** 

**Supplementary Material 2.** 

**Supplementary Material 3.** 

## Data Availability

The datasets used and/or analysed during the current study are available from the corresponding author on reasonable request.

## Abbreviations

BM: Bone marrow
β2M: Beta-2-microglobulin
BTZ: Bortezomib
[Ca2+]_o_: Extracellular calcium
CCK-8: Cell Counting Kit-8
CQ: Chloroquine
IDA: Iron deficiency anemia
IOD: Integrated optical density
ISS: International Staging System
MM: Multiple myeloma
NCX1: Na^+^-Ca^2+^ exchanger 1
OS: Overall survival
SD: Standard deviation
TBS: Tris-buffered saline
TEM: Transmission Electron Microscopy
TRPV4: Transient receptor potential vanilloid 4
PI: Proteasome inhibitors
PVDF: Polyvinylidene difluoride
UPR: Unfolded protein response
