# Supplementary figures and images for: NCX1/Ca2+ promotes autophagy and decreases bortezomib activity in multiple myeloma through non-canonical NFκB signaling pathway
Source: Cell Commun Signal. 2024 May 6;22:258. doi: 10.1186/s12964-024-01628-4 (PMC11075190; doi:10.1186/s12964-024-01628-4)

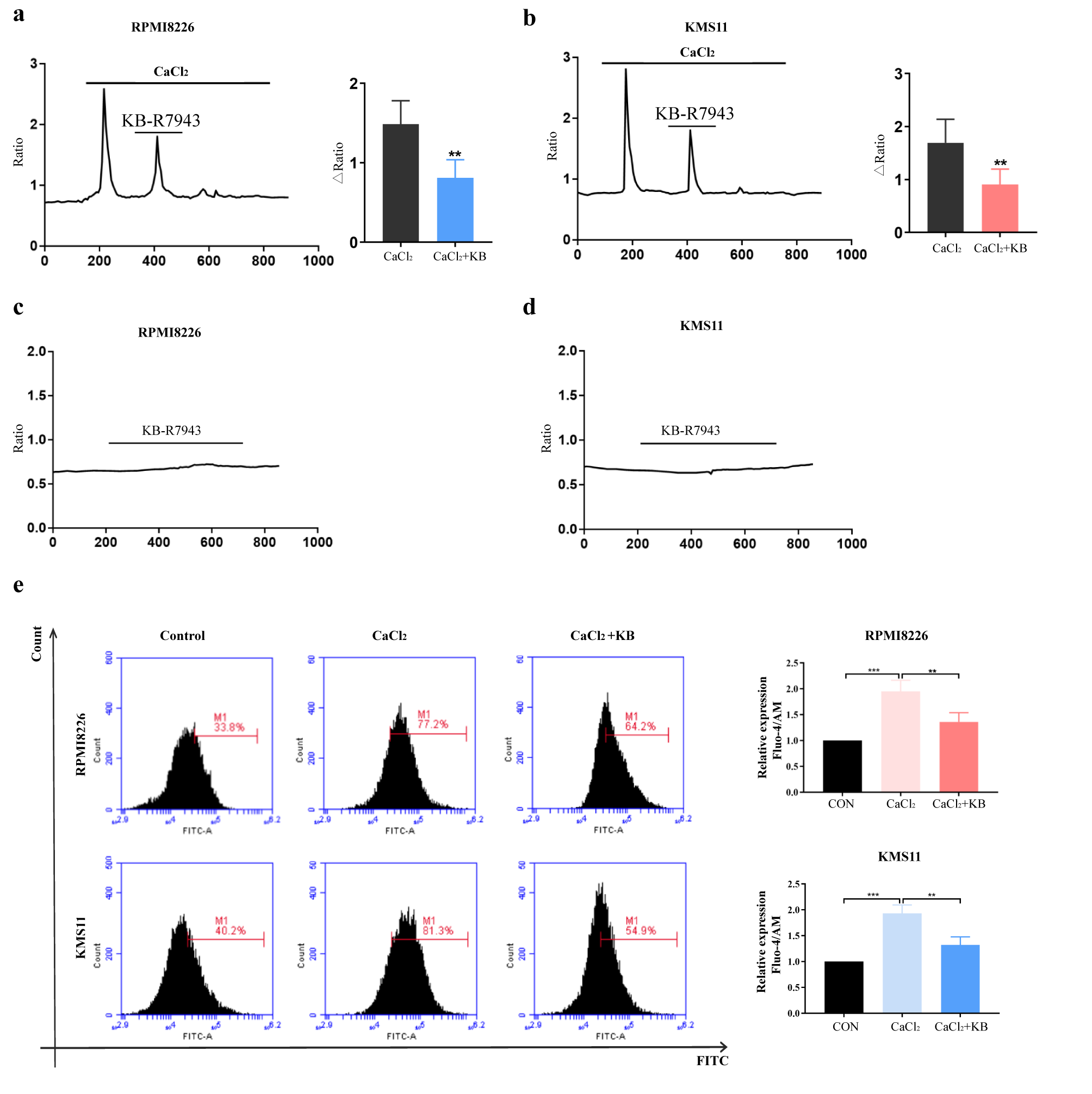

Supplement: Supplementary file 1 — Supplementary Material 1. [file 12964_2024_1628_MOESM1_ESM.tif]

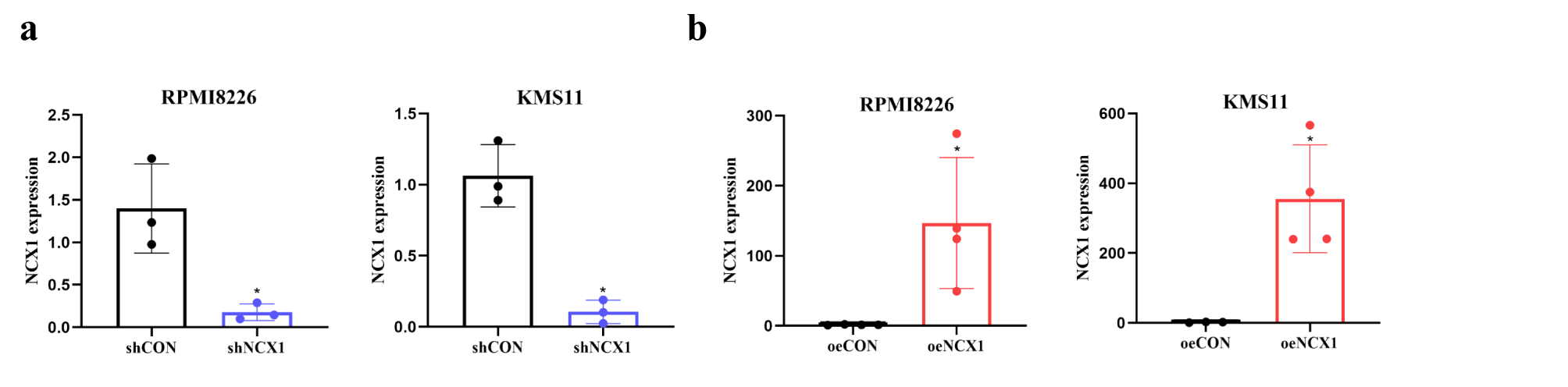

Supplement: Supplementary file 2 — Supplementary Material 2. [file 12964_2024_1628_MOESM2_ESM.tif]

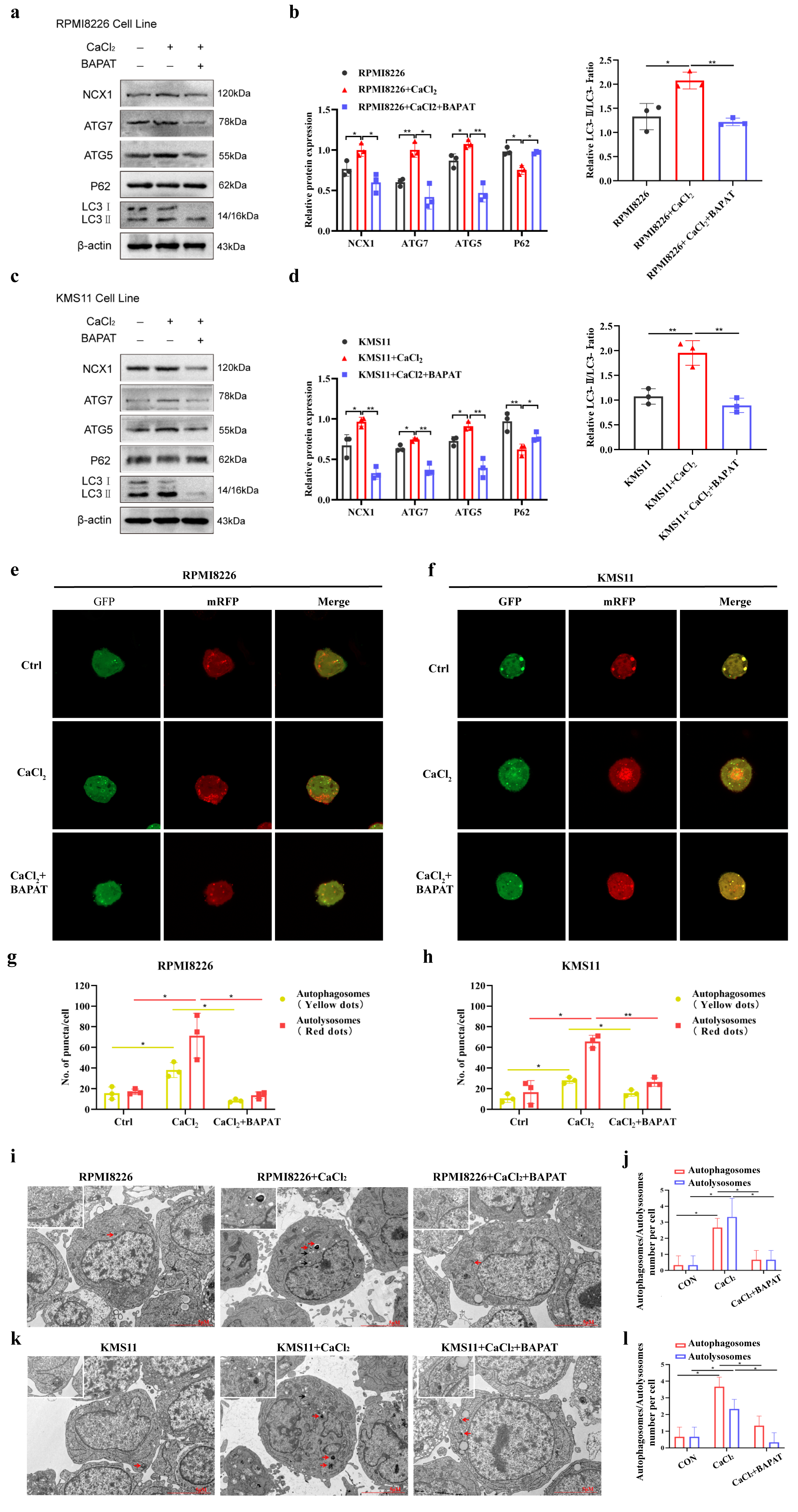

Supplement: Supplementary file 3 — Supplementary Material 3. [file 12964_2024_1628_MOESM3_ESM.tif]
